# Digestion

**Published:** 1869-02

**Authors:** John W. Holt


### ARTICLE II.

### *Digestion.*

By John W. Holt, D.D.S.

*Concluded.*

It has been observed that during the intervals of digestion, the secretion of the gastric juice, if not entirely suspended, is extremely scanty. Food however, is the natural stimulus of the digestive apparatus, and when naturally ingested, the stomach ordinarily responds with promptness and vigor. The flow of the gastric juice commences within a few minutes after the food is swallowed, and continues until stomach digestion is completed. The time varies as the nature of the food. According to Dr. Beaumont's experiments, *pigs feet*, or *tripe*, may be digested in an hour, while *roasted pork* requires more than five hours for digestion.

Prof. Dalton estimates that in a man, nearly fourteen pounds of gastric juice is secreted daily ; from which we may presume that the average quantity for an ordinary meal is more than *four pounds*. Probably however, there is never more than a few ounces of the fluid in the stomach at once, for absorption progresses with secretion and digestion, and as fast as the gastric juice takes the aliment in solution, it passes into the circulation.

The peristaltic muscular action of the stomach is another important circumstance of digestion. The action is excited by the presence of the food, and continues until the stomach is emptied. It is a gentle churning motion which affects the thorough admixture of the aliment with the gastric juice, and expels the chyme through the pyloric orifice into the small intestine, where the digestion of the albuminoid substances, commenced in the stomach, is continued, and the other ingredients of the food are digested.

In the small intestine, the chyme from the stomach, meets with three different secretions, viz: the true *intestinal juice*, secreted by the follicles of Lieberkhun, and the duodenal glandulæ of Bruner; the bile from the liver, and the secretion of the pancreas. Physiologists have experimented on intestinal digestion by means of fistulæ, but with results less satisfactory than the observations in regard to the stomach, for the several fluids, as secreted under natural circumstances, are mixed with each other, and with the gastric secretion. It seems to be decided, however, that the secretions of the duodenal glands and follicles, the intestinal juice proper, digests sugar and starch, for their common product, *glucose*, is found in the duodenum, above the orifice of the biliary and pancreatic ducts. The transformation of these substances commence immediately after they pass the pyloric orifice, and the process is remarkably rapid. Prof. Dalton found that in a dog, a full meal of boiled starch was not only digested, but entirely absorbed, in three-quarters of an hour. It is, perhaps even more rapid in the human subject.

The intestinal juice is clear, colorless and viscid. Its reaction is decidedly alkaline. The quantity found in the intestine by experimenters is very scanty, and insufficient for extensive experiments.

The oily matters, as we have seen, are unchanged in the stomach, only set free by the disintegration of the tissues with which they may be entangled, and melted by the heat. In the duodenum, however, below the orifice of the biliary and pancreatic ducts, the oily drops are rapidly broken up and reduced to a white, milky emulsion called *chyle*. It was somewhat difficult to decide which, of the fluids poured into the intestine, effects this reduction, and various opinions have existed, but experiments by M. Bernard and others, show conclusively, that the digestion of the oleaginous substances is due to the pancreatic juice.

The pancreatic juice is a clear, colorless, slightly viscid, alkaline fluid, with a specific gravity of about 1008 or 1009. The analysis by Bidder and Schmidt gives the composition as follows;

| | |
|---|---|
| Water, . . . . . . . | 900.76 |
| Organic matter, (Pancreatine.) . . | 90.38 |
| Chloride of sodium, . . . . . | 7.36 |
| Salts of soda, potassa, lime, magnesia &c. | 1.50 |
| | 1000.00 |

Prof. Dalton estimates the quantity of pancreatic juice secreted by an average man, at 1.872 pounds daily. The secretion in the intervals of digestion is inconsiderable.

The action of the pancreatic juice on the oily substances, is prompt and decided, the chyle appearing immediately after the contact. Unlike the digestion of the other ingredients of the food however, the fatty matters are believed to suffer no chemical alteration, not even solution, but are only reduced by contact with the *pancreatine*, to a state of minute subdivision, in which condition it is capable of being absorbed.

Bile, the secretion of the liver, is a dark brown, viscid fluid, with a specific gravity of about 1018. Nearly 90 per cent. of its composition is water, besides which, it contains a fatty substance, coloring matter, cholesterin, mucus, and various salts of soda, potassa, lime and magnesia. Its reaction is alkaline. The quantity secreted daily is estimated at nearly two and a-half pounds, for an average man.

Physiologists have held varions opinions in regards to the functions of the bile, but there seems to be very little really known about it. On one hand the facts, that it is secreted constantly, regardless of digestion: that unlike other secretions of the body, it is produced from venous blood: that if retained and taken up by the circulation, it stains the animal tissues and secretions, and produces symptoms of poisoning &c., would lead us to regard the bile as simply excrementitious, and unnecessary, as regards digestion, especially since we have already provided for the digestion of all of the ingredients of the food. On the other hand however, if we consider the situation of the liver and biliary duct, in relation with the organs of digestion; and the facts that the bile, though present constantly, is poured into the intestine most abundantly during digestion, and that like the digestive fluids before noticed, the greater part of it, is absorbed from the intestinal canal, with the aliment, and not passed off with the feces, we are forced to believe that it is, in some manner, subservient to digestion, absorption, or the nutritive process: and experiments by Prof. Flint and others, prove decidedly that such is the fact. A dog with an artificial fistula, through which the bile, secreted normally, was discharged freely, but excluded from the intestine, soon died from inanition: from which experiment we conclude that the bile is not only essentially excrementitious, but recrementious and histogenetic. In regard to the nature of the influence exercised by the bile, however, very little is known. It is believed that it neutralizes the fatty acids, saponifies the fats, and assists the pancreatic juice in emulsifying them; that it stimulates the action of the intestinal glands, and the peristaltic muscular action; that by virtue of an antiseptic property, it prevents putrefactive decomposition of the contents of the intestine, &c. The bile looses its indentity by the process of digestion, for though it is undoubtedly absorbed, it is not found in the blood of the portal vein.

Digestion, we find then, includes several different processes. The food is masticated and mixed with the saliva in the mouth: in the stomach it meets the gastric juice, by which the tissues are disintegrated, the sugar, starch and fats set free, and the albuminoid substances converted into albuminose: in the small intestine, the sugar and starch are transformed into glucose, by contact with the intestinal juice, and the oily matter is reduced to an emulsion, by the pancreatic fluid. Digestion however, thus distinctly commenced by the several secretions, is continued simultaneously in the intestine, and thus all of the ingredients of the food, with their respective digestive fluids, mingled together, are subjected to the influence of the bile, whatever that may be. As the aliment passes downward by the vermicular action of the intestine, digestion progresses. All of the digestive parts of the food are gradually disintegrated, their nutrient materials liquefied and absorbed with the several secretions. Consequently, as the contents reach the lower part of the small intestine, they gradually become more consistent, and darker in color: and finally in the large intestine, where digestion and absorption are completed, the mass gradually assumes the coherent consistency, and other characters of the feces, and contains little more than the indigestible, fibrous and horny parts of the food, epithelial scales and cells, mucus, coloring matter of the bile, phosphate of lime, magnesia, soda &c., and the indefinite excrementitious substance, peculiar to the large intestine. The feces are expelled occasionally, as their presence in the rectum, excites the contraction of its muscular walls, and the abdominal muscles.

